# Hepatic steatosis relates to gastrointestinal microbiota changes in obese girls with polycystic ovary syndrome

**DOI:** 10.1371/journal.pone.0245219

**Published:** 2021-01-19

**Authors:** Beza Jobira, Daniel N. Frank, Lori J. Silveira, Laura Pyle, Megan M. Kelsey, Yesenia Garcia-Reyes, Charles E. Robertson, Diana Ir, Kristen J. Nadeau, Melanie Cree-Green

**Affiliations:** 1 Department of Pediatrics, Division of Pediatric Endocrinology, University of Colorado Anschutz Medical Campus, Aurora, Colorado, United States of America; 2 Department of Medicine, Division of Infectious Diseases, University of Colorado Anschutz Medical Campus, Aurora, Colorado, United States of America; 3 Department of Biostatistics and Informatics, Colorado School of Public Health, Aurora, Colorado, United States of America; 4 Center for Women’s Health Research, University of Colorado Anschutz Medical Campus, Aurora, Colorado, United States of America; East Tennessee State University, UNITED STATES

## Abstract

**Objective:**

Hepatic steatosis (HS) is common in adolescents with obesity and polycystic ovary syndrome (PCOS). Gut microbiota are altered in adults with obesity, HS, and PCOS, which may worsen metabolic outcomes, but similar data is lacking in youth.

**Methods:**

Thirty-four adolescents with PCOS and obesity underwent stool and fasting blood collection, oral glucose tolerance testing, and MRI for hepatic fat fraction (HFF). Fecal bacteria were profiled by high-throughput 16S rRNA gene sequencing.

**Results:**

50% had HS (N = 17, age 16.2±1.5 years, BMI 38±7 kg/m^2^, HFF 9.8[6.5, 20.7]%) and 50% did not (N = 17, age 15.8±2.2 years, BMI 35±4 kg/m^2^, HFF 3.8[2.6, 4.4]%). The groups showed no difference in bacterial α-diversity (richness p = 0.202; evenness p = 0.087; and diversity p = 0.069) or global difference in microbiota (β-diversity). Those with HS had lower % relative abundance (%RA) of *Bacteroidetes* (p = 0.013), *Bacteroidaceae* (p = 0.009), *Porphyromonadaceae* (p = 0.011), and *Ruminococcaceae* (p = 0.008), and higher *Firmicutes*:*Bacteroidetes (F*:*B)* ratio (47.8% vs. 4.3%, p = 0.018) and *Streptococcaceae* (p = 0.034). Bacterial taxa including phyla *F*:*B* ratio, *Bacteroidetes*, and family *Bacteroidaceae*, *Ruminococcaceae* and *Porphyromonadaceae* correlated with metabolic markers.

**Conclusions:**

Obese adolescents with PCOS and HS have differences in composition of gut microbiota, which correlate with metabolic markers, suggesting a modifying role of gut microbiota in HS and PCOS.

## Introduction

Polycystic Ovary Syndrome (PCOS) is a common endocrine disorder among women of reproductive age. Its prevalence is approximately 6–12%, affecting at least 5 million women in the U.S [1–3], depending upon the diagnostic criteria used. PCOS is characterized by hyperandrogenism, with the clinical presentation including acne, hirsutism, and irregular menses [1–4]. PCOS is often accompanied by obesity [5], and is associated with higher risk for type 2 diabetes (T2D), cardiovascular disease, nonalcoholic fatty liver disease (NAFLD), infertility, pregnancy complications, and depression [1, 3, 6, 7].

Approximately 10% of U.S. children ages 2–19 years have NAFLD, and the majority of these youth are overweight or obese. NAFLD comprises a range of disease stages including simple steatosis, nonalcoholic steatohepatitis (NASH), cirrhosis, and hepatocellular carcinoma [8]. The development of NASH is multifactorial relating to factors including fat deposition, oxidative stress, Toll-like-receptor-mediated signaling [9], adipose-tissue derived signaling [10], endoplasmic reticulum stress [11], and genetic factors [12]. The majority of NAFLD cases remain as simple steatosis while 25% progress to NASH, liver fibrosis and ultimately cirrhosis [13]. About 10% of liver transplants are currently secondary to NASH in the U.S., and NASH is projected to be the most common indication for transplantation in the near future [14]. NAFLD is also a significant contributor to the risk for developing T2D, a condition which has a greatly increased prevalence in women with PCOS [15].

NAFLD prevalence is higher in girls and adult women with PCOS compared to women of similar BMI without PCOS. For example, a higher prevalence of NAFLD is reported in adult women with PCOS, compared to equally obese adult women [16]. Similarly, we previously demonstrated that in obese adolescent girls with PCOS, the NAFLD prevalence is 50%, compared to only a 13% prevalence in equally obese adolescent girls without PCOS [17]. It thus appears that women with PCOS have a unique risk for NAFLD, beyond obesity.

The gut microbiota has been shown to play a role in the pathogenesis of both PCOS and NAFLD [18, 19]. Alterations in four predominant gastrointestinal phyla (*Firmicutes*, *Bacteroidetes*, *Proteobacteria*, *Actinobacteria*) have been associated with high fat/low fiber diet, obesity, insulin resistance, T2D, PCOS and NAFLD [1–3, 7, 20–23]. We previously demonstrated that youth with PCOS have a unique microbiota profile relative to similarly obese girls without PCOS [24]. We thus sought to examine the gut microbiota composition in adolescent girls with combined obesity and PCOS, with or without HS, to determine if there is a unique microbiota profile associated with HS, beyond alterations seen with obesity and PCOS status.

## Methods

### Participants

A total of 34 participants with PCOS were included from 2 separate cross-sectional studies (APPLE NCT02157974, N = 18; PLUM NCT03041129, N = 16) if they completed stool collection. Participants were recruited from the Pediatric Endocrinology and Lifestyle Medicine outpatient clinics at Children’s Hospital Colorado. Inclusion criteria were female sex, age 12–20 years, overweight/obesity (BMI >90%ile), Tanner stage 5, post-menarchal status, and sedentary status (<3 hours of habitual physical activity/week; validated with a 3-day physical activity recall). Exclusion criteria were BP >140/90 mmHg, hemoglobin <9 mg/dL, serum creatinine >1.5 mg/dL, smoking, medication affecting insulin sensitivity (oral steroids, metformin, thiazolidinediones, atypical antipsychotics, hormonal contraceptives), antihypertensive medications, statins, pregnancy, breast feeding, and any antibiotic use in the previous 2 months. The NIH criteria with adolescent adaptation were used to define PCOS: oligomenorrhea defined as <8 menses per year, clinical or biochemical signs of hyperandrogenism and at least 18 months post menarche [25].

### Study approval

The study protocols were approved by the University of Colorado Institutional Review Board and the Children’s Hospital of Colorado Scientific Advisory Review Committee. Informed written consent or assent was obtained from all participants as appropriate for age, and parental written consent from all participants <18 years of age.

### Study protocol

Participants had a screening visit for consent, physical exam and laboratory measurements to confirm eligibility. They then underwent a 2 day study visit which included stool collection, DEXA and abdominal MRI, followed by a monitored 12-hour inpatient fast with morning fasting blood collection and then an oral sucrose tolerance test consisting of 75 grams of glucola and 25 grams of fructose, survey completion and another physical exam. Waist circumference, BMI (kg/m^2^), and BMI percentile per Center for Disease Control and Prevention BMI growth charts [26] were obtained. Abdominal MRI was used to assess hepatic fat via the DIXON method of the entire liver, as previously described [27] HS was defined as HFF ≥ 5.0%. Hepatic stiffness was assessed with MR elastography. Total body fat percentage was assessed by standard DEXA methods (Hologic, Waltham, MA).

### Physical activity

A 3-day pediatric activity recall (3DPAR) questionnaire was completed with staff assistance from all participants to assess habitual physical activity [28].

### Dietary intake

A diet interview by study staff was completed using the SEARCH food frequency questionnaire (FFQ) to assess macronutrient patterns. The FFQ is defined to incorporate and represent common food choices among ethnically and regionally diverse youth aged 10–19 years [29].

### Laboratory measurements

Fasting glucose, sex hormone concentrations, inflammatory markers and lipid profiles were measured. Glucose was measured by a StatStrip hospital grade glucometer (Nova Biomedical, Waltham, MA). Serum insulin and adiponectin were analyzed with RIA (Millipore, Billerica, MA); FFA (Wako Chemicals, Inc., Richmond, VA) were assessed enzymatically. HbA1c was measured by DCCT-calibrated ion-exchange HPLC (Bio-Rad Laboratories, Hercules, Calif). Alanine aminotransferase (ALT) and aspartame aminotransferase (AST) were measured by multipoint rate with P-5-P method (Vitros^®^ 5600, Ortho Clinical Diagnostics, Raritan, NJ); total cholesterol, high density lipoprotein cholesterol (HDL-C), and triglyceride assays were performed enzymatically on a Hitachi 917 autoanalyzer (Boehringer Mannheim Diagnostics, Indianapolis, IN). Low density lipoprotein cholesterol (LDL-C) concentrations were calculated by the Friedewald equation; highly sensitive C-reactive protein (hs-CRP) was measured via immunoturbidimetric assay (Beckman Coulter, Brea, CA), C-peptide via chemiluminescent immunoassay (DiaSorin, Stillwater, MN), and estradiol and progesterone via chemiluminescent immunoassay (Beckman Coulter, Brea, CA). Total testosterone was measured by high-pressure liquid chromatography/tandem mass spectrometry, free testosterone via equilibrium dialysis and sex hormone binding globulin (SHBG) via chemiluminescent immunoassay, all by Esoterix laboratories (Calbassas Hills, CA). Hepatic fat fraction (HFF) was measured by MRI as previously described [17] and hepatic stiffness was measured by MR elastography. DXA was used to measure percent body fat and lean mass as previously described [30].

### Fecal collection and microbiome analysis

Stool samples were collected at home the day prior to blood sampling using stool collection tubes and frozen in the participant’s freezer. Upon return to study staff, samples were stored at -80°C until further processing. Bacterial profiles were determined by broad-range analysis of 16S rRNA genes following our previously described methods [24, 31]. In brief, DNA was extracted from 50–100 mg of stool using the PowerFecal DNA isolation kit (QIAamp Powerfecal DNA kit (Qiagen INC, Hilden, Germany). Broad-range PCR amplicons were generated using barcoded primers targeting the V3V4 variable region of the 16S rRNA gene: primers 338F (5’ ACTCCTACGGGAGGCAGCAG) and 806R (5’ GGACTACHVGGGTWTCTAAT). PCR products were normalized using a SequalPrep^™^ kit (Invitrogen, Carlsbad, CA) and paired-end sequencing performed on the Illumina MiSeq platform using a 600-cycle version 3 reagent kit. 16S rRNA gene sequences were demultiplexed, quality filtered, culled of human and chimeric sequences [32], and classified using the SINA/SILVA platform [33, 34] as previously described [24, 31]. Operational taxonomic units (OTUs) were produced by clustering sequences with identical taxonomic assignments. Between 34,307 to 201,471 sequence reads were generated per sample and Good’s coverage was >99.0% for all samples.

### Calculations

Insulin sensitivity was estimated using the homeostasis model assessment-of insulin resistance [HOMA-IR = (FG * FI) / (405), where FI = fasting insulin μU/mL and FG = fasting glucose (mg/dL)] [35] and by the Matsuda index [10,000/ √(FG*FI)/(mean G*mean I)] [36]. Four participants with HS and five without received a one-time dose of a glucose modulating medication following stool collection and were not included in the HOMA-IR and Matsuda index calculations.

### Statistical analysis

Data analyses were performed using R version 3.5.2 and Sigmaplot version 13.0. Data were examined for normality. Differences between the groups were compared with students t-tests or Mann-Whitney U, as appropriate. For categorical data either Fisher’s exact tests or Pearson chi-square tests were performed to test differences between groups. The %RA of each taxon was calculated as the number of 16S rRNA sequences of a given taxon divided by the total number of 16S rRNA sequences in a patient’s sample. Differences in overall microbiome composition (β-diversity) between subsets were assessed by a non-parametric, permutation-based multivariate analysis of variance (PERMANOVA with 10,000 replicate re-samplings) using Morisita-Horn dissimilarities. Shannon diversity, Shannon evenness, and richness (Sobs) (measures of α-diversity) were calculated using rarefaction and compared across groups using linear models adjusting for batch effects [37]. Comparisons of %RA across groups were performed using Wilcoxon rank sum tests since batch and race/ethnicity effects were not significant in any of the individual phyla, family or genus comparisons. Spearman’s correlations were used to evaluate the relationship between %RA and metabolic and hormonal variables. Bacterial taxa with %relative abundance > 1% were used for correlations and markers of insulin resistance, obesity, fatty liver disease were used as variables. Results were adjusted for age, race/ethnicity and protein intake. The correlations were adjusted for multiple testing and those with p-values ≤0.05 were reported.

## Results

### Clinical characteristics

Thirty-four girls completed the baseline assessment and returned the stool sample and thus were included in final analyses. The group was equally split into those with or without HS (n = 17 for each). Participant demographic and physical characteristics and laboratory measurements are summarized in Table 1. The groups had similar age, age of menarche, and family histories of type 2 diabetes. There was however a significant difference in race/ethnicity across groups, with more Hispanic representation in the HS group. Physical characteristics including BMI, waist-to-hip ratio and blood pressure were similar across groups. Both groups reported a similar percentage of dietary fat, protein and carbohydrate intake, and habitual physical activity.

**Table 1:** Cohort characteristics.

	Hepatic Steatosis, N = 17	No Hepatic Steatosis, N = 17	p-value
**Demographics and Family History**			
Age (years)	16.2 ± 1.5	15.8 ± 2.2	0.52
Race (Caucasian Black, Asian %)	82, 18, 0	71, 17, 12	**0.01**
Ethnicity (Hispanic, Non-Hispanic)	65, 35	12, 88	**0.001**
Menarche age (years)	11.9 ± 1.2	11.2 ± 1.2	0.09
Family history of T2D (%)	82	76	1.0
**Physical Characteristics**			
BMI (kg/m^2^)	38 ± 7	35 ± 4	0.19
BMI (%ile)	99 (97, 99)	98.1 (97, 99)	0.35
BMI Z-score	2 ± 0.4	2 ± 0.3	0.49
Waist-to-hip ratio	0.9 ± 0.1	0.9 ± 0.5	0.08
Hepatic fat fraction	9.8 (6.5, 20.7)	3.8 (2.6, 4.4)	**<0.001**
Hepatic stiffness (kPa)	2.6 ± 0.3	2.6 ± 0.5	0.77
Total body fat by DEXA (%)	45.4 ± 3.7	45.2 ± 2.9	0.92
Lean body mass by DEXA (%)	52.2 ± 3.6	52.2 ± 2.9	0.97
Systolic BP (mmHg)	123 (116, 129)	124 (120, 136)	0.56
Diastolic BP (mmHg)	72 (69, 78)	69 (65, 71)	0.10
**7-day Dietary Intake Recall**			
Fat intake (%)	36 (30, 43)	41 (37, 44)	0.27
Protein intake (%)	15 ± 2.4	17 ± 2.2	0.08
Carbohydrate intake (%)	48 (37, 57)	42 (38, 47)	0.21
**Physical Activity**			
Activity from recall survey (METS)	53 ± 8	54 ± 9	0.83
**Laboratory Measurements**			
AST (IU/mL)	45 (39, 72)	41 (34, 51)	0.19
ALT (IU/mL)	45 (33, 49)	32 (30, 38)	**0.003**
WBC (10^9^ cells/L)	7.8 ± 1.1	8.7 ± 2.1	0.13
Platelets (10^8^ cells/L)	317 (288, 362)	328 (301, 358)	0.65
hs-CRP (mg/dL)	3.6 (1.1, 7.6)	2.4 (1.1, 6.4)	0.73
Adiponectin (ng/mL)	6.2 (4.6, 7.1)	6.5 (4.9, 10.8)	0.51
Triglycerides (mg/dL)	120 (100, 184)	107 (83, 145)	0.39
Cholesterol (mg/dL)	140 (134, 177)	145 (129, 168)	0.99
HDL (mg/dL)	32 (29, 45)	36 (32, 45)	0.52
LDL (mg/dL)	108 (92, 140)	108 (95, 123)	0.92
HbA1c (%)	5.7 ± 0.2	5.4 ± 0.4	**0.02**
Fasting glucose (mg/dL)‡	92 ± 9	86 ± 8	0.12
Fasting insulin (μU/mL) ‡	30 (22, 49)	19 (14, 31)	**0.03**
Fasting C-peptide (ng/mL) ‡	3.0 ± 0.8	2.1± 0.9	**0.01**
Two hour glucose (mg/dL) ‡	140 ± 32	135 ± 26	0.68
Two hour insulin (μU/mL) ‡	193 (167, 708)	152 (35, 271)	0.08
HOMA-IR‡	8.3 ± 4	5.4 ± 3.4	0.07
Matsuda Index‡	1.0 (0.6, 1.5)	2.0 (1.1, 2.7)	**0.02**
Free testosterone (ng/dL)	9.2 (7.3, 13.5)	8.2 (6.7, 11.0)	0.29
Total testosterone (ng/dL)	40 (30.0, 58.5)	43 (29.5, 50.5)	0.78
SHBG (mmol/L)	18 (11, 22)	21 (13, 29)	0.17
Estradiol (pg/mL)	53 (45, 77)	56 (44, 107)	0.37
Progesterone (ng/dL)	0.5 (0.4, 0.7)	0.6 (0.5, 1.5)	0.08

Values are mean ± standard deviation of the mean, or median (25%ile, 75%ile). BMI = Body Mass Index. T2D = Type 2 Diabetes. METS = Metabolic Equivalents. SHBG = sex hormone binding globulin. HDL = high density lipoprotein. LDL = low density lipoprotein. HgA1c = hemoglobin A1c. AST = aspartate transferase. ALT = alanine transferase. hsCRP = highly sensitive c-reactive protein. HOMA-IR = homeostatic assessment insulin resistance. Only ‡N = 13 for HS had glucose measurements and only ‡N = 12 for no-HS had glucose measurements.

The groups had similar free and total testosterone, SHBG, estradiol and progesterone. Per study design, girls with HS had significantly higher HFF compared to those without HS, as well as ALT. There were no group differences in AST, hepatic stiffness or body composition. Girls with HS had higher fasting insulin and C-peptide, and HbA1c and were more insulin resistant (higher HOMA-IR and lower Matsuda index) than those without HS. There were no differences in fasting glucose, 2-hour glucose or insulin, triglycerides, total cholesterol, HDL-C, LDL-C or adiponectin between groups. Markers of inflammation including WBC, platelets, and hs-CRP were also similar between groups.

### Dysbiosis in hepatic steatosis

**Fig 1:**
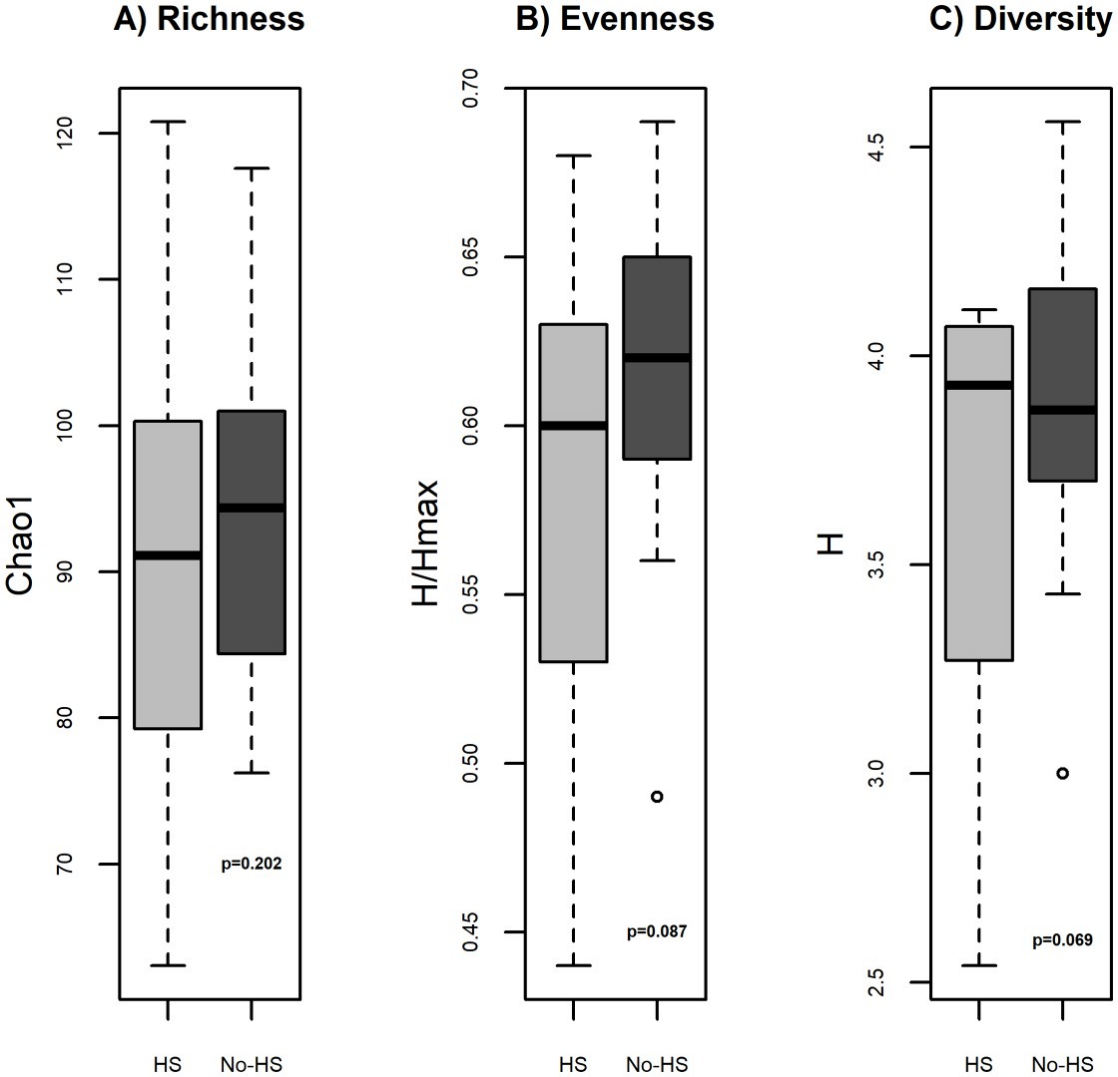
Alpha biodiversity measures. Measures of α-biodiversity including: A) Richness (Chao1) B) Evenness (Shannon H/Hmax), C) Shannon diversity (Shannon H). There were no statistical differences in α- and β-diversity between groups.

**Fig 2:**
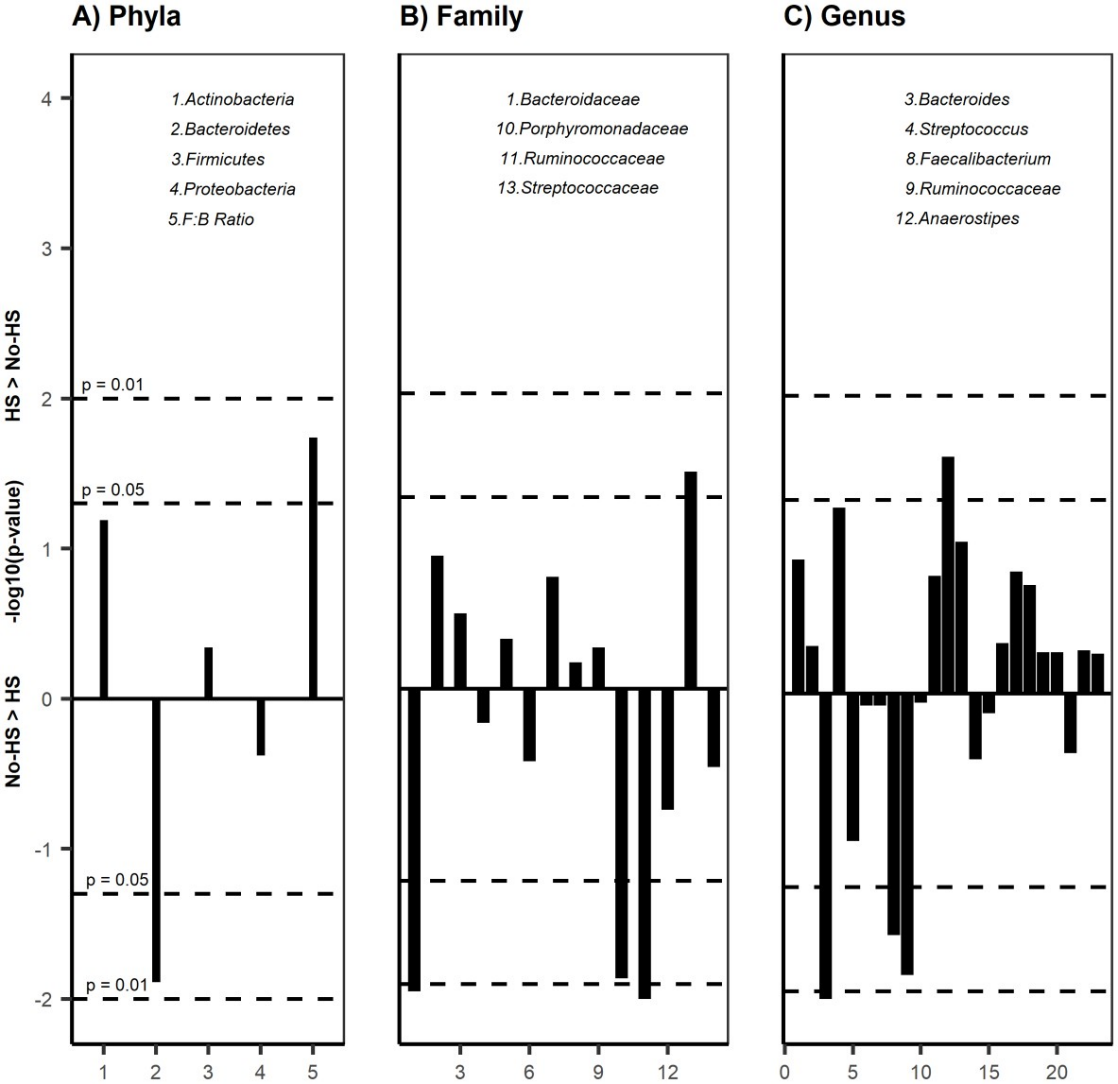
Percent relative abundance in HS and No-HS. Manhattan plot of bacterial %RA between groups at the A) Phyla B) Family and C) Genus level. Only taxa with > 1% RA are included. Lines above 0 are >%RA in HS and below 0 >%RA in No-HS, with dotted horizontal lines representing p<0.05 and p<0.01. HS had higher %RA of *Firmicutes*:*Bacteroidetes* ratio and lower *Bacteroidetes*. At the family level, HS had higher %RA of *Streptococcaceae*, *and lower Bacteroidaceae*, *Porphyromonadaceae*, and *Ruminococcaceae*. At the genus level, HS had higher %RA of *Streptococcus* and *Anaerostipes*, and lower %RA of *Bacteroides*, *Faecalibacterium* and *Ruminococcaceae*.

Bacterial 16S rRNA gene profiling was completed for all samples; both groups had adequate depth of sequencing coverage (Good’s coverage of >99.0% for all samples) indicating comparable and representative samples. Girls with HS had numerically but not statistically significantly measures of alpha diversity including lower bacterial richness (p = 0.202) and evenness (p = 0.087) and higher diversity (p = 0.069) (Fig 1) compared with those without HS. The β-diversity, reflecting overall gut microbial community composition, was similar between groups (R^2^ = 0.036, p = 0.35). There were still no differences in α- and β-diversity measures after adjusting for group differences in race/ethnicity, age, and protein intake percentage. *Actinobacteria* and *Firmicutes* were the most predominant phyla in those with HS, and *Firmicutes* and *Bacteriodetes* were the most dominant phyla in girls without HS. At the phylum level, girls with HS had significantly lower percent relative abundance (%RA) of *Bacteroidetes* and higher *Firmicutes*:*Bacteroidetes* (*F*:*B*) ratio (47.8% vs. 4.3%, p = 0.018) than those without HS. At the family level, girls with HS had lower %RA of *Bacteroidaceae* (p = 0.009), *Porphyromonadaceae* (p = 0.011), *Ruminococcaceae* (p = 0.008), and higher *Streptococcaceae* (p = 0.034) than those without HS. Fig 2 depicts the comparison of %RA at the phyla, family, and genus levels.

### Bacterial taxa are associated with metabolic markers

Several taxa correlated with hepatic steatosis and markers of the metabolic syndrome as shown in Table 2. A higher *F*:*B* ratio was correlated with more central adiposity (higher waist-to-hip ratio), higher ALT and HFF, and insulin resistance as assessed by two-hour OSTT insulin. A lower %RA of *Bacteroidetes*, *Bacteroidaceae*, *Porphyromonadaceae*, and *Ruminococcaceae* were correlated with HFF. A lower %RA of *Ruminococcaceae* was correlated with higher triglycerides.

**Table 2:** Correlations between clinical measurements and percent relative abundance of bacterial taxa.

Variables	Phyla or family	R value	P-value*
**Waist-to-hip ratio**	*Firmicutes*:*Bacteriodetes* ratio (P)	0.433	**0.050**
	*Bacteriodetes (P)*	-0.404	0.083
	*Bacteroidaceae (F)*	-0.442	0.060
**Systolic blood pressure**	*Streptococcaceae (F)*	0.437	0.069
**ALT**	*Firmicutes*:*Bacteriodetes* ratio (P)	0.417	**0.050**
	*Bacteriodetes (P)*	-0.386	0.084
	*Bacteroidaceae (F)*	-0.406	0.060
**Hepatic fat fraction**	*Firmicutes*:*Bacteriodetes* ratio (P)	0.526	**0.020**
	*Bacteriodetes (P)*	-0.529	**0.018**
	*Bacteroidaceae (F)*	-0.536	**0.015**
	*Porphyromonadaceae (F)*	-0.535	**0.016**
	*Ruminococcaceae (F)*	-0.465	**0.044**
**Triglycerides**	*Ruminococcaceae (F)*	-0.459	**0.044**
**Fasting insulin**	*Ruminococcaceae (F)*	-0.405	0.094
**Two-hour insulin**	*Firmicutes*:*Bacteriodetes* ratio (P)	0.508	**0.050**
	*Bacteriodetes (P)*	-0.507	0.083
	*Ruminococcaceae (F)*	-0.455	0.082
**Matsuda index**	*Bacteriodetes (P)*	0.433	0.086
	*Firmicutes*:*Bacteriodetes* ratio (P)	-0.442	0.075
	*Bacteroidaceae (F)*	0.474	0.060
	*Ruminococcaceae (F)*	0.477	0.074
	*Streptococcaceae (F)*	-0.533	0.069
**HOMA-IR**	*Ruminococcaceae (F)*	-0.401	0.094

*Bacterial taxa associated with variables with a p-value <0.1 are reported. P-values are adjusted for multiple comparisons. (P) = phyla, (F) = family.

## Discussion

The gut microbiome is different in individuals with either PCOS or NAFLD, as compared to controls. We have demonstrated the novel finding that adolescents with PCOS, obesity and HS have an altered gastrointestinal microbiota compared to those with PCOS and obesity without HS. Significant differences were noted in the %RA of several phyla, families, and genera by HS status, and these bacterial taxa were significantly correlated with multiple metabolic markers related to NAFLD and insulin resistance. While there were some non-significant group differences in α-diversity, the β-diversity, which indicates global microbial community alteration between groups, these did not differ by HS status. Thus, it appears that the addition of HS beyond obesity and PCOS status is associated with changes in specific microbiota, but not overall global changes in the microbiome.

Our findings are consistent with results in adolescents and adult women with either PCOS or NAFLD, though there are limited data in adolescent girls with both PCOS and NAFLD. Zhu et al. demonstrated that ecological differences (α-diversity and β-diversity) in the gut microbiota among adolescent girls and boys (age 12–14 years) are related to health status, obesity, and NASH [38]. Another study by Chierico et al. found a significant difference in β-diversity when comparing normal weight healthy youth controls to those diagnosed with obesity without NAFLD, HS alone, and NASH. However, this study also found no difference in β-diversity between youth with obesity without NAFLD, HS alone, and NASH [39]. The combination of these findings and ours suggest that obesity may potentially play a role in influencing diversity measures, though we did not have normal weight group for comparison to confirm this.

Relative abundance of specific bacteria can vary with obesity and NAFLD status. For example, adolescents with NASH and obesity had predominantly *Prevotella*-rich microbiota [40], whereas non-obese, non-NASH groups were more frequently associated with *Bacteroides* rich enterotypes [38]. In contrast, we found that girls with PCOS, obesity and HS had lower %RA of the family *Prevotellaceae*, but had lower %RA of *Bacteroides*. Zhu et al. found a statistically significant decrease in *Bacteroidetes* and an increase in *Firmicutes* in a non-obese adolescent group when compared to adolescents with simple obesity and to those with NASH. We found a statistically significantly lower amount of *Bacteroidetes* in those with HS and no difference in *Firmicutes* between groups. The study by Chierico et al. in boys and girls (mean age, 10–12 years) comparing non-obese control youth to youth with obesity, NAFLD, and NASH alone, found that those with NAFLD had higher proportion of *Actinobacteria* and Proteobacteria compared to NASH, obese, and healthy control, and reduced *Bacteroidetes* and *Firmicutes* compared to youth with obesity [39]. This study also found that participants with NAFLD and NASH had gut microbiota signatures with an increase in %RA of *Ruminococcus* and *Dorea*, whereas we found lower %RA of *Ruminoccocaeae* in our HS cohort. The differences seen in our patient population compared to non-PCOS NAFLD patients could potentially reflect changes influenced by age, PCOS status, local dietary patterns and only including female sex participants. Future provocative interventional studies would be needed to confirm if these findings of association do indeed have mechanistic underpinnings.

The pathophysiological link between PCOS and NAFLD remains unclear [16, 41]; however, insulin resistance and obesity are common critical components in both NAFLD and PCOS [8, 13, 42]. There is also evidence that insulin resistance and hyperandrogenism mediate the relationship between PCOS and NAFLD [43]. Studies have demonstrated that hyperandrogenic women with PCOS had higher liver fat compared with women with PCOS based on the Rotterdam criteria with normal androgens or with healthy controls [44]. We found significant correlations between HFF and several bacterial families and phyla, suggesting a relationship between HFF and the gut microbiome, but not androgen concentrations. Another study demonstrated that women with PCOS and NAFLD had decreased hepatic LDL receptor expression, and hypothesized that hyperandrogenism may putwomen with PCOS at risk for development of dyslipidemia and NAFLD [45]. Although we did not find correlations between LDL-C and bacterial taxa, we had several taxa that correlated with characteristics of the metabolic syndrome and with a marker of insulin resistance, suggesting that the gut microbiota may relate to increased risk of T2D, NASH, and cardiovascular disease. Additionally, alcohol producing bacteria may contribute to the pathogenesis of NAFLD in PCOS. For example, Zhu et al. found that ethanol metabolism and *Enterobacteriaceae* have a functional relationship in contributing to the development of NASH [38]. Adolescent patients with NASH were also found to have upregulation of ethanol metabolism compared to controls [39]. In addition to *Enterobacteriaceae*, *Bacteroides*, *Bifidobacterium* and *Clostridium* are also alcohol producing bacteria [38]. Though we did not measure blood or breath alcohol concentration, we found higher %RA *Bifidobacterium* in our adolescents with HS, which suggests that bacterial taxa involved in ethanol production may contribute to endogenous ethanol production in NALFD in PCOS.

Limitations to our study include the small sample size and lack of blood and breath alcohol tests, which may have provided further understanding of NASH pathogenesis in girls with PCOS. HOMA-IR, the Matsuda index and insulin values were used to estimate insulin sensitivity, rather than a gold-standard hyperinsulinemic euglycemic clamp and thus we were also not able to assess tissue specific of insulin sensitivity. It is also unknown if our participants had NASH or liver fibrosis as we did not perform liver biopsy, although MR elastography results do not indicate notable stiffness. The groups were not matched for ethnicity, with a greater proportion of Hispanics in the NAFLD group, consistent with a higher prevalence of NAFLD in those with Hispanic origin. We attempted to mitigate this group difference by adjusting analysis for race/ethnicity status, but it is possible that there is a reflection of underlying increased risk for NAFLD in this group. We can only comment on associations between measures, since our study design was not longitudinal, and thus our findings are hypotheses generating for future provocative studies on causation. There are several unique strengths to our study. Our groups were similar in terms of age, age of menarche, pubertal stage, BMI, diet and physical activity and PCOS markers. Further, liver fat of the entire liver was measured using MRI instead of using liver ultrasound or relying on only laboratory liver enzymes. Finally, we used the NIH criteria to define PCOS, which identifies a more metabolically at-risk population.

## Conclusion

In girls with obesity and PCOS, the composition of the gut microbiota is different in those with HS compared to those without HS. In this cohort, HS was associated with alterations in the gut microbiota that are typically related to metabolically unhealthy obesity. Furthermore, in the overall cohort, certain taxa at the phylum and family level were correlated with insulin resistance, and the metabolic syndrome characteristics of central adiposity, and elevated triglycerides showing a relationship between the gut microbiota and increased risk of T2D, NASH, and cardiovascular disease. Our findings suggest that there is a relationship between the gut microbiome and metabolic disease in adolescents with HS and PCOS, but it remains unclear which components come first and whether the relationships are causative or just associations. Further work is warranted to better understand the pathogenesis of HS and PCOS, the role of the gut microbiota in adolescence and to potentially develop therapies in the future to help reduce risk of T2D, cardiovascular disease and liver disease.
